# Green solvents and Ultrasound-Assisted Extraction of bioactive orange (*Citrus sinensis*) peel compounds

**DOI:** 10.1038/s41598-019-52717-1

**Published:** 2019-11-06

**Authors:** Abigail Montero-Calderon, Clara Cortes, Ana Zulueta, Ana Frigola, Maria J. Esteve

**Affiliations:** Department of Nutrition and Food Chemistry, University of Valencia, Avda. Vicent Andrés Estellés, s/n, 46100 Burjassot, Spain

**Keywords:** Sustainability, Characterization and analytical techniques

## Abstract

Byproducts such as orange peel have potential uses because of their bioactive compounds, which are important for their potential to reduce the risk factors of diseases caused by aging. The lack of effective techniques and the high levels of pollution produced by the conventional extraction of bioactive compounds using organic solvents have highlighted the need to enhance the ‘green chemistry’ trend. This study evaluates the use of ultrasound to extract bioactive compounds from orange peel. The antioxidant capacity, phenolic content, ascorbic acid, total carotenoids, and HPLC profile of phenolic compounds from orange peel extracts were obtained by a physicochemical evaluation. The results demonstrate that the optimal conditions for the ultrasound-assisted extraction of bioactive orange peel compounds were a power of 400 W, a time of 30 min, and 50% ethanol in water. These conditions were used to obtain a total carotenoid concentration of 0.63 mg ß-carotene/100 g, vitamin C concentration of 53.78 mg AA/100 g, phenolic concentration of 105.96 mg GAE/100 g, and antioxidant capacity of ORAC = 27.08 mM TE and TEAC = 3.97 mM TE. The major phenolic compound identified in all orange peel extracts was hesperidin, with a maximum concentration of 113.03 ± 0.08 mg/100 g.

## Introduction

The current food trends around the world are aligned with the healthy and sustainable consumption of natural products, such as fruits and vegetables. An example of this is the Slow Food movement, which aims to counteract the spread of fast food^1^. The World Health Organization (WHO) recommends a minimum intake of 600 g/person/day of fruits and vegetables because their vitamins, carotenoids, and polyphenols can prevent ischemic heart disease, cerebrovascular disease, and cancer of the stomach, colon, esophagus, and lung^2,3^.

In this context, orange has important nutritional components, such as amino acids, carbohydrates, fiber, proteins, vitamins, and minerals^4^. This citrus is historically the most consumed fruit (77.3 g/person/day) and has been experiencing a favorable evolution of consumption since 2000^5^. In 2013, the total orange production in Spain reached 3.536.745 tonnes^6^.

As food consumption has increased, its waste has increased, which has negative implications for natural resources conservation^7^. Food waste is a solid or liquid substance that is deliberately or unintentionally discarded during the processing, preparation, storage, handling, or selling of food. Organic waste, such as fruit peel, is also considered food waste^8^. In 2013, the European Union set the objective of reducing 50% of its food waste by 2020^9^. In 2015, the United States proclaimed the national goal of reducing half the food waste and loss of food by 2030^7^.

As reported by Park *et al*.^10^ reported that compounds extracted from fruit residues can be used in functional and nutraceutical foods because of their antioxidant content. Citrus residues are the seeds, remnants of the pulp after squeezing the juice, and peel, which comprises the albedo and flavedo^11^. Vitamin C and ß-carotene are abundant compounds in the pulp and peel of citrus^3^; flavones, flavanones, chalcones, and dihydrochalcones are flavonoids characteristic of the genus Citrus^12^.

The lack of effective techniques for processing citrus residues to obtain a quality raw material suitable for new products is one of the fundamental reasons that this matrix is not leveraged in the industry. Similarly, the low efficiency and environmental pollution produced by the organic solvents used in conventional extraction have highlighted the need to enhance the trend known as ‘green chemistry’ and develop methods that produce less pollution and achieve the high-performance extraction of bioactive compounds in a short time and at low cost. The most frequently used techniques in clean extraction are ultrasound-assisted extraction (UAE), microwaves, and extraction with supercritical fluids^12^.

UAE is based on the application of ultrasounds (20–100 kHz) that cause the implosion of the cavitation bubbles of cells on which the acoustic waves are propagated, leading to the disruption of cell membranes. This action facilitates the penetration of solvent into the cells, thereby improving mass transfer and releasing bioactive compounds. The performance of phenolic compound extraction using UAE has been verified^13^, but the solvents used in the verification produce serious environmental pollution. Therefore, it is necessary to continue studying the optimization of bioactive compound extraction using ‘green solvents’ such as ethanol and water. To this end, the aim of this study is to obtain bioactive compounds from orange peel with an extraction method that combines UAE and environmentally friendly solvents.

## Results and Discussion

### The effect of ultrasound extraction on physicochemical properties

Several parameters (pH, degrees Brix, conductivity, and color) of the extracts were determined. All orange extracts had an acidity pH between 4.91 ± 0.00 and 5.97 ± 0.01, while Irkin *et al*.^14^ reported a pH of 6.62 ± 2.2 for orange peel extracts in their investigation.

Degrees Brix increased as ultrasonic power, extraction time, and ethanol concentration increased (17.05 ± 0.07 °Bx; 400 W, 30 min, and 50% ethanol). Legua *et al*.^15^ obtained values from 12.2 to 14.2 °Bx for tangerine juice and expressed that the determination of °Bx is related to the soluble solids content in a solution, especially sugars. The increase in °Bx correlates with the intensity of the ultrasonic treatment applied, which means an increase in the breakage of plant cells and the diffusion of the content into the extraction liquid. In the same way, the highest conductivity value obtained was 0.45 ± 0.14 mS/cm with 400 W, 30 min, and 0% ethanol. The conductivity represents the electrolytic content of the extracts of orange peel, therefore, it shows the exit from inside the plant cells.

The colors of the extracts ranged between brown and yellow and are represented by the following values: L*: 5.75 ± 0.03 to 40.24 ± 0.02; a*: −1.15 ± 0.01 to 4.15 ± 0.05; and b*: 2.47 ± 0.01 to 18.63 ± 0.03. The main extraction of pigments (ΔE*) was obtained for the high value of ultrasound power (400 W). A similar result was reported for the characterization of commercial orange nectar (L* 39.5 ± 3.00; a* −3.72 ± 0.90; b* 16.4 ± 4.80) by Alvarez *et al*.^16^, who emphasized the importance of color for the acceptability of a product because it is the first visible quality.

### Determination of antioxidant capacity

ORAC analysis obtained values between 4.35 ± 0.39 (100 W, 5 min, 0% ethanol) and 29.23 ± 2.38 mM TE (400 W, 5 min, 50% ethanol). These results are higher than those obtained by Park *et al*.^10^ of 0.006 mM TE for orange peel extracts obtained through conventional extraction using acetone.

For TEAC, the values were from 0.89 ± 0.07 mM TE (100 W, 5 min, 0% ethanol) to 3.97 ± 0.15 mM TE (400 W, 30 min, 50% ethanol), which are higher than the value obtained by Mhiri *et al*.^17^ for orange peel extracts using UAE (0.003 mM TE; 125 W, 30 min, 80% ethanol). The increase in antioxidant capacity is due to an increase in compounds with antioxidant capacity, because the cavitation phenomena cause the breakage of plant walls.

ANOVA results show that ultrasound power (P), ultrasound irradiation time (T), and the concentration of ethanol in water (E) had a significant effect (p < 0.05) on the antioxidant capacity of orange peel extracts. The experimental data are in accordance with the second-order polynomial equations expressed as follows:

$$\begin{array}{rcl}{\rm{ORAC}}({\rm{mM}}\,{\rm{TE}}) & = & 0.080\ast {\rm{P}}+0.485\ast {\rm{T}}+0.063\ast {\rm{E}}\\  &  & -\,0.0001\ast {{\rm{P}}}^{2}-\,0.001\ast {\rm{P}}\ast {\rm{T}}+0.001\\  &  & \ast \,{\rm{P}}\ast {\rm{E}}+0.005\ast {\rm{T}}\ast {\rm{E}}-5.286\end{array}$$

(R^2^ = 87.03, p < 0.05, standard error = 2.85)

$$\begin{array}{rcl}TEAC(mM\,TE) & = & 1.774-0.012\ast P+0.022\ast T\\  &  & +0.039\ast E+2.5\ast {10}^{-5}\ast {P}^{2}+0.0001\ast \\  &  & \,P\ast T-0.001\ast {E}^{2}\end{array}$$

(R^2^ = 75.68, p < 0.05, standard error = 0.38)

where R^2^ indicates that the models predict 87.03% and 75.68% of the results obtained by ORAC and TEAC, respectively.

### Vitamin C

The minimum and maximum ascorbic acid values obtained were 8.89 ± 1.54 and 93.33 ± 2.67 mg AA/100 g; these results are in some agreement with the concentration of 99 mg AA/100 g obtained by Cano and Bermejo^18^ from orange albedo extract (*C*. *sinensis* L. cv. Navelina). ANOVA results show that ultrasound power (P), ultrasound irradiation time (T), the power–time interaction (P*T), and time–ethanol interaction (T*E) are the main factors (p < 0.05) that affect ascorbic acid extraction from orange peel according to the following model:

$$AA(\frac{mg}{100g})=-\,10.632-0.005\ast P+1.879\ast T+0.004\ast P\ast T-0.029\ast T\ast E$$

(R^2^ = 70.45, p < 0.05, standard error = 13.67)

### Total carotenoids

The high carotenoid value obtained was 0.63 ± 0.01 mg ß-carotene/100 g; in some experiments, it was not detected (ND). The results for the total carotenoid content of orange peel are usually variable; in a study by Fidrianny *et al*.^19^, the carotenoids reached 21 mg ß-carotene/100 g from the extraction of orange peel with 100% ethanol. The ANOVA results show that only the extraction time and percentage of ethanol have a statistically significant influence (p < 0.05) on carotenoid extraction. The contribution of the experimental factors to CT values are represented by the following equation:

$$CT(\frac{mg}{100g})=0.143-0.01\ast T+0.004\ast E$$

(R^2^ = 53.60, p < 0.05, standard error = 0.11)

### Total soluble phenols

TSP content ranged from 16.01 ± 1.59 to 105.96 ± 1.92 mg GAE/100 g. There are diverse results because of the variety of citrus, extraction conditions, and solvents. Kour *et al*.^11^ determined a phenolic content of 17.1 ± 1.04 mg GAE/g in mandarin residues, Park *et al*.^10^ found 1.39 mg GAE/100 g in orange peel, Irkin *et al*.^14^ obtained 1108 mg GAE/100 g from orange peel, and Fidrianny *et al*.^19^ determined that the average content in the peel of three varieties of orange was 9.49 mg GAE/100 g with conventional extraction using ethanol.

**Figure 1:**
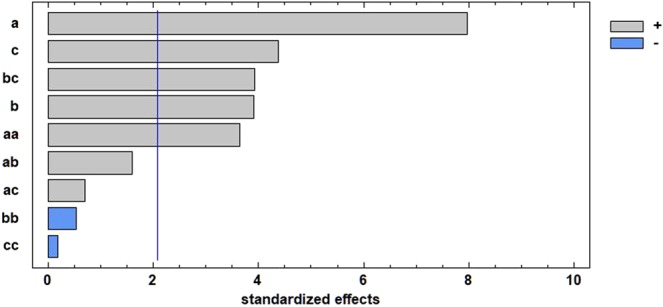
Pareto diagram for the extraction of TSP from orange peel. a = ultrasound power (W), b = ultrasound irradiation time (min), c = concentration of ethanol in water (%).

Linear effects, quadratic effects, and interactions between experimental factors were statistically significant for the extraction of TSP from orange peel (Fig. 1). The horizontal bars represent the positive and negative effects of the experimental factors on the response variable, while the vertical line indicates the significance of these effects at a 95% confidence level.

There are five statistically significant effects in UAE of phenols from orange peel: the linear effect of ultrasound power (P), the linear effect of ethanol percentage (E), the quadratic effect of the time–ethanol interaction (T*E), the linear effect of extraction time (T), and the quadratic effect of ultrasound power (P^2^). The effects are described by the following model:

$$\begin{array}{ccc}TSP(\frac{mg}{100g}) & = & 45.889-0.273\ast P-0.194\ast T\\  &  & -\,0.058\ast \,E+0.001\ast {P}^{2}+0.029\ast \,T\ast E\end{array}$$

(R^2^ = 86.57, p < 0.05, standard error = 9.20)

### Characterization of phenolic compounds

**Figure 2:**
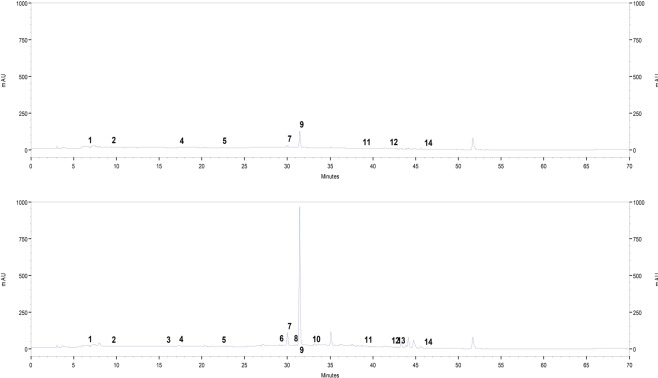
Chromatographic profile for orange peel extracts using UAE for the ultrasonic power, extraction time, and ethanol concentration of 100 W, 5 min, and 0% ethanol (**a**) and 400 W, 30 min, and 50% ethanol (**b**). (1) Gallic acid, (2) protocatechuic acid, (3) (+)-catechin, (4) caffeic acid, (5) p-coumaric acid, (6) chlorogenic acid, (7) ferulic acid, (8) naringin, (9) hesperidin, (10) rutin trihydrate, (11) trans-cinnamic acid, (12) quercetin, (13) apigenin, and (14) hesperetin.

Two chromatograms corresponding to the minimum and maximum experimental factors are shown in Fig. 2. The major phenolic compound identified in all orange peel extracts was hesperidin, with a maximum concentration of 113.03 ± 0.08 mg/100 g. Irkin *et al*.^14^ emphasized that the high antioxidant capacity of orange peel is associated with its high content of hesperidin.

The maximum concentrations of protocatechuic acid and trans-cinnamic acid were 1.43 ± 0.05 and 0.09 ± 0.00 mg/100 g, respectively. Karoui and Marzouk^20^ found higher concentrations of these phenols (2 mg/100 g) for bitter orange peel extracts, but the value of catechin (4 mg/100 g) found by the authors was lower than the maximum determined in the present study (9.15 ± 0.25 mg/100 g).

The values of ferulic acid (0.38 ± 0.00 to 7.42 ± 0.13 mg/100 g), chlorogenic acid (ND to 0.82 ± 0.02 mg/100 g), hesperidin (14.60 ± 0.05 to 113.03 ± 0.08 mg/100 g), p-coumaric acid (0.06 ± 0.00 to 2.07 ± 0.03 mg/100 g), and naringin (ND to 0.35 ± 0.01 mg/100 g) were compared with the values obtained for the peel of a hybrid orange citrus (*Citrus sinenses* (L) × *Citrus unshiu* Marc.) by He *et al*.^21^, who obtained a ferulic acid concentration of 2.74 mg/100 g, chlorogenic acid concentration of 1.39 mg/100 g, and hesperidin concentration of 265.75 mg/100 g; p-coumaric acid and naringin were not detected.

The concentrations of caffeic acid (0.02 ± 0.02 to 2.11 ± 0.02 mg/100 g), gallic acid (0.09 ± 0.00 to 2.95 ± 0.01 mg/100 g), rutin (ND to 2.83 ± 0.35 mg/100 g), and quercetin (ND to 0.19 ± 0.00 mg/100 g) are in some agreement with the results for orange peel extracts obtained by Irkin *et al*.^14^, who obtained a caffeic acid concentration of 0.019 mg/100 g and did not detect gallic acid, rutin, and quercetin.

Finally, the values of apigenin (ND to 0.12 ± 0.00 mg/100 g) and hesperetin (0.14 ± 0.00 to 1.20 ± 0.01 mg/100 g) differ from those in the study by Menichini *et al*.^22^ for citrus peel (*Citrus medica* L. cv Diamante). The main flavonoids found in their study were apigenin (6.28 mg/100 g) and hesperetin (5.04 mg/100 g).

### Optimal extraction conditions

Response surface methodology was used in order to evaluate interactions between experimental conditions, and the desirability function was determined to generate optimum conditions with a desirability value.

The maximum extraction of bioactive compounds from orange peel was achieved with the highest values of the experimental factors. Zou *et al*.^23^ determined that the optimal percentage of ethanol in water corresponded to 50% for the extraction of bioactive compounds from *Mangifera indica* leaves.

**Figure 3:**
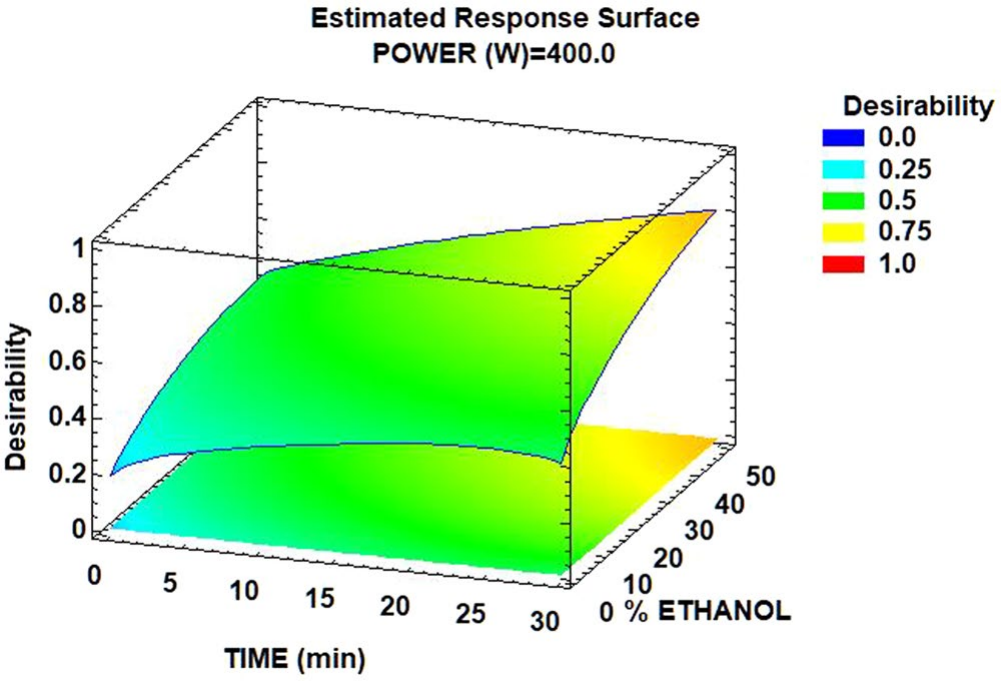
Surface response for the ultrasound-assisted extraction of orange peel compounds.

The optimal conditions determined using Statgraphics were an ultrasonic power of 400 W, an ultrasound irradiation time of 30 minutes, and a concentration of ethanol in water of 50%. The combination of factors at which the optimum response was achieved generated a desirability value of 0.794 (Fig. 3).

Under optimal conditions, the maximum response values predicted were a carotenoid concentration of 0.52 mg ß-carotene/100 g, vitamin C concentration of 63.20 mg AA/100 g, TSP concentration of 94.82 mg GAE/100 g, ORAC of 28.48 mM TE, TEAC of 3.61 mM TE, and ΔE* of 26.16. There was a real efficiency of the assay because the experimental results were consistent with the predictive values. The experiment using 400 W, 30 min, and 50% ethanol resulted in a carotenoid concentration of 0.63 ± 0.01 mg ß-carotene/100 g, vitamin C concentration of 53.78 ± 3.36 mg AA/100 g, TSP concentration of 105.96 ± 1.92 mg GAE/100 g, ORAC of 27.08 ± 2.17 mM TE, TEAC of 3.97 ± 0.15 mM TE, and ΔE* of 28.87 ± 0.03.

## Conclusions

As expected, the optimum extraction conditions of bioactive orange peel compounds using UAE corresponded to the maximum tested values of ultrasonic power, extraction time, and percentage of ethanol in water. It is not studied to increase the percentage of ethanol for the extraction, because our objective was to obtain an extraction with reduced resources (less use of solvents). The established conditions obtained a TC concentration of 0.63 mg ß-carotene/100 g, vitamin C concentration of 53.78 mg AA/100 g, TSP concentration of 105.96 mg GAE/100 g, ORAC of 27.08 mM TE, and TEAC of 3.97 mM TE.

The results of this study suggest that it is possible to use UAE as a non-conventional technique for the extraction of bioactive compounds from orange residues. Moreover, environmentally friendly solvents were used for the extraction without altering the physicochemical parameters of the samples. Finally, response surface methodology was accurate in its determination of the optimum conditions of extraction since the predicted and experimental values were similar.

## Methods

### Sample

Navel orange (*Citrus sinensis* Osb.) was obtained from the Valencian Community. The albedo and flavedo were separated from the edible parts of the fruit, and then the orange peel was cut into squares, each side equal to 0.6 cm, using a regular vegetable chopper (Lacor S.L., España).

### Chemicals

HCl (hydrochloric acid), 2,6-DCFI (2,6-dichloroindophenol), FCR (Folin–Ciocalteu reagent), K_2_S_2_O_8_ (potassium persulfate), ethanol, and gallic acid were purchased from Panreac (Barcelona, Spain). Na_2_HPO_4_ (sodium hydrogen phosphate), KH_2_PO_4_ (potassium dihydrogen phosphate), and NaHCO_3_ (sodium hydrogen carbonate) were obtained from Scharlab (Barcelona, Spain). Na_2_CO_3_ (sodium carbonate), acetone, acetonitrile, and ascorbic acid were provided by VWR International (Lovaina, Belgium). Formic acid was purchased from Merck (Darmstadt, Germany). Acetic acid, hexane, and methanol were supplied by J.T. Baker (Deventer, The Netherlands). HPO_3_ (metaphosphoric acid), Trolox ((+/−)-6-hydroxy-2,5,7,8-tetramethylchromane-2-carboxylic acid), ABTS (2,2′-azino-bis(3-ethylbenzothiazoline-6-sulphonic acid)), AAPH (2,2′-azobis (2-methyl)propionamidine), fluoresceine, protocatechuic acid, (+)-catechin, caffeic acid, chlorogenic acid, p-coumaric acid, ferulic acid, trans-cinnamic acid, naringin, hesperetin, hesperidin, and apigenin were obtained from Sigma-Aldrich (St. Louis, USA). Rutin trihydrate and quercetin were obtained from HWI Analytik GmbH (Ruelzheim, Germany). Analytical grade chemicals and distilled water were used.

### Extraction procedure

Orange peel squares were placed in a beaker with ethanol–water in a ratio of 1:10 (g/mL)^24^ as the solvent. The beaker was immersed in ice to prevent the extract from exceeding 40 °C during ultrasonication; the temperature was controlled by a thermometer Testo 925 (Testo, Germany). The extraction was carried out in the ultrasonic processor Q500 (Qsonica, USA), and the extraction conditions were set as follows: ultrasound power was set to 100, 250, and 400 W; ultrasound irradiation time was 5, 17.5, and 30 min; and the concentration of ethanol in water was 0%, 25%, and 50%.

### Physicochemical parameters

The physicochemical parameters of orange peel extracts were determined. Two measurements of each sample were taken. The conductivity was measured using an LF330 conductivity meter (Wissenschaftlich-Technische Werkstätten, Germany); pH and °Bx were measured according to IFU^25^ with MicropH 2001 (Crison, Spain) and Master-T (Atago, Japan), respectively. The color was measured with a ColorQuest XE equipment (HunterLab, USA) considering the CIELAB (Commission Internationale de l’Eclairage LAB) system, and non-UAE orange peel extract was used to determine ΔE* as the total color difference^26^.

### Determination of antioxidant capacity

#### ORAC (Oxygen radical absorbance capacity) assay

The total antioxidant capacity of the extracts obtained by UAE was evaluated according to the procedure described by Ou *et al*.^27^ with the modifications to reagent concentrations made by Zulueta *et al*.^28^. Fluorescein was added to a white microtiter plate (Sterilin Limited, UK); AAPH, Trolox, and orange peel extracts were added to selected wells to compare the fluorescein degradation. The final reactions were measured using a Wallac 1420 VICTOR2 multilabel counter (Perkin Elmer, USA).

#### TEAC (Trolox equivalent antioxidant capacity) assay

The TEAC assay shows the capacity of a sample to inhibit the ABTS radical (ABTS• + ) compared with the antioxidant standard Trolox. The method was described by Re *et al*.^29^ with modifications by Zulueta *et al*.^28^ for the final reaction tested. The ABTS stock solution was prepared by mixing 25 mL of ABTS (7 mM) with 440 µL of K_2_S_2_O_8_ (140 mM) and incubating the solution in the dark for 16 h to obtain a stable ABTS working solution for mixing with diluted orange extract (sample/ethanol, 1:25, v/v). The absorbance was measured using a UV/VIS Lambda 2 spectrophotometer (Perkin Elmer, USA) at λ = 734 nm and equilibrated with a Julabo UC-5B circulator thermostat (Julabo, Germany) at 30 °C. Two measurements of each sample were taken.

### Determination of bioactive compounds

Ascorbic acid, total carotenoids, total phenolic compounds, and phenolic characterization of orange peel extracts were determined by analyzing duplicate samples.

#### Ascorbic acid

The ascorbic acid (AA) content in orange peel extracts was determined by redox titration^30^. The ascorbic acid standard was diluted (50%) with an extraction solution (metaphosphoric acid–acetic acid, 50:50, v/v) and titrated with 2.6 DCFI. The same procedure was used for the samples of orange peel extract. The results are expressed in mg of AA per 100 g of orange peel.

#### Total carotenoids

The total carotenoids (TC) in the samples were determined using the spectrophotometric method by Lee and Castle^31^. Orange peel extract (2 mL) with extracting solvent (hexane/acetone/ethanol, 50:25:25, v/v) (5 mL) was centrifuged for 5 min at 4000 rpm and 5 °C in a 5810 R centrifuge (Eppendorf, Germany). The supernatant was diluted (1:1, v/v) with hexane, and the absorbance was measured at 450 nm on a UV/VIS Lambda 2 spectrophotometer (Perkin Elmer, USA). For determining the concentration of total carotenoids, the extinction coefficient of ß-carotene E^1%^ = 2505 was used in accordance with Ritter and Purcell^32^. The results are expressed in mg of ß-carotene per 100 g of orange peel.

#### Total soluble phenols

Total soluble phenols (TSP) were determined according to the colorimetric method of Singleton and Rosi^33^ with the modifications of George *et al*.^34^. For the standard curve, 0.1 mL of gallic acid calibration standard with concentrations of 0–1000 ppm were placed in tubes with 0.1 mL of Folin–Ciocalteau reagent (1:1, v/v) and 3 mL of sodium carbonate solution (2%, w/v) and incubated for 60 min at room temperature in darkness. The same procedure was used for orange peel extracts diluted with distilled water (1:1, v/v). TSP determination was carried out by measuring the absorbance at λ = 765 nm. The results are expressed in mg of gallic acid equivalents (GAE) per 100 g of sample.

### Characterization of phenolic compounds

A quantitative analysis of phenolic compounds was performed by using high-performance liquid chromatography (HPLC) in accordance with Cichova *et al*.^35^. The analytical system used was an Agilent 1120 Compact LC (Agilent Technologies, Germany) with a UV 280 nm detector and equipped with a 4.6 × 250 mm column Luna C18, 100 Å, 5 µm (Phenomenex, USA). The flow rate was 1 mL/min, the temperature of the column oven was 25 °C, and the injection volume was 20 µL. The mobile phase consisted of water–formic acid (95:5, v/v) as solvent A and acetonitrile–solvent A (60:40, v/v) as solvent B. A gradient program was carried out as follows: 0 min, 100% A; 10 min, 85% A; 20 min, 82% A; 50 min, 0% A; 65 min, 100% A; 70 min, 100% A.

The analytical standards were prepared at a stock concentration of 200 μg/mL. The preparation of the sample before running the HPLC auto-sampler consisted of injecting 5 mL of methanol and 10 mL of water in a Sep-Pak C18 cartridge (Phenomenex, USA); then, 5 mL of orange peel extract, adjusted to pH 1.6, was passed through the cartridge. Phenolic compounds were collected with 12 mL of acetone, which was then evaporated with nitrogen and dissolved with 1 mL of HCl 0.6 M/aqueous methanol 75%. The results are expressed in mg per 100 g of orange peel.

### Experimental design and statistical analysis

A central composite design (CCD) was used to evaluate the effects of independent variables (ultrasound power: 100, 250, and 400 W; ultrasound irradiation time: 5, 17.5, and 30 min; and concentration of ethanol in water: 0%, 25%, and 50%) on the dependent variables (color, antioxidant capacity, ascorbic acid, carotenoids, and TSP). The selection criteria for the levels of experimental factors levels included the operating conditions of the UAE equipment and the characteristics of the sample.

CCD is an experimental design used to obtain the maximum information in a study from a minimal number of experiments. Further, CCD is a suitable standard design that can be used in response surface methodology (RSM) because of its high efficiency and the number of runs required. The type of CCD used was the central composite face-centered (CCF) design, in which the start points are placed at the center of each face of the factorial space (α = ±1). CCF was performed with the combinations shown in Table 1 at three levels (maximum, central, and minimum: +1, 0, −1) of each independent variable.

**Table 1:** Experimental design matrix of CCD in terms of variables for experiments on UAE for orange peel.

Run	*Ultrasound power* W	*Time* Min	*Ethanol in water* %
	(*X*_1_)	(*X*_2_)	(*X*_3_)
1	400	5	50
2	400	17.5	25
3	250	17.5	0
4	400	5	0
5	400	30	0
6	100	5	0
7	400	30	50
8	250	17.5	25
9	100	17.5	25
10	100	30	50
11	100	5	50
12	250	17.5	25
13	250	17.5	50
14	100	30	0
15	250	30	25
16	250	5	25

During execution, the experiments were randomized to increase accuracy and minimize systematic bias. Once the different treatments of samples were carried out in duplicate, the optimal settings of the experimental quantitative factors were identified. Analysis of variance (ANOVA) was performed in order to test the statistical significance (p < 0.05) of the effects by comparing the mean square with an estimate of the experimental error. In this way, the test helped to find the best design points to obtain a combination of the low and high levels of each factor with the highest predicted desirability.

RSM was applied to determine the optimal conditions of the significant factors of extraction and to obtain a predictive model that describes changes in the response depending on ultrasound power, ultrasound irradiation time, and the concentration of ethanol in water. RSM involves the simultaneous effects of the three levels of each experimental factor and establishes a response variable as a linear function of experimental factors, interactions of factors, error, and quadratic effects.

The desirability function was used on a scale of 0 to 1 to determine the combination of levels of independent variables that maximize the desirability of values of the dependent variables.

The experimental design generation and statistical methods were performed using the software Statgraphics Centurion XVI 15.2.06 (Statpoint Technologies Inc., USA).

## Data Availability

The datasets generated during and/or analysed during the current study are available from the corresponding author on reasonable request.
